# Toward transplantation tolerance with adipose tissue-derived therapeutics

**DOI:** 10.3389/fimmu.2023.1111813

**Published:** 2023-04-28

**Authors:** Hui-Yun Cheng, Madonna Rica Anggelia, Cheng-Hung Lin, Fu-Chan Wei

**Affiliations:** ^1^ Center for Vascularized Composite Allotransplantation, Chang Gung Memorial Hospital at Linkou, Taoyuan, Taiwan; ^2^ Department of Plastic and Reconstructive Surgery, Chang Gung Memorial Hospital at Linkou, Taoyuan, Taiwan; ^3^ School of Medicine, Chang Gung University, Taoyuan, Taiwan

**Keywords:** transplantation, tolerance, stromal vascular fraction (SVF), secretome, AD-MSCs

## Abstract

Solid organ and composite tissue allotransplanation have been widely applied to treat end-stage organ failure and massive tissue defects, respectively. Currently there are a lot of research endeavors focusing on induction of transplantation tolerance, to relieve the burden derived from long-term immunosuppressant uptake. The mesenchymal stromal cells (MSCs) have been demonstrated with potent immunomodulatory capacities and applied as promising cellular therapeutics to promote allograft survival and induce tolerance. As a rich source of adult MSCs, adipose tissue provides additional advantages of easy accessibility and good safety profile. In recent years, the stromal vascular fraction (SVF) isolated from adipose tissues following enzymatic or mechanical processing without *in vitro* culture and expansion has demonstrated immunomodulatory and proangiogenic properties. Furthermore, the secretome of AD-MSCs has been utilized in transplantation field as a potential “cell-free” therapeutics. This article reviews recent studies that employ these adipose-derived therapeutics, including AD-MSCs, SVF, and secretome, in various aspects of organ and tissue allotransplantation. Most reports validate their efficacies in prolonging allograft survival. Specifically, the SVF and secretome have performed well for graft preservation and pretreatment, potentially through their proangiogenic and antioxidative capacities. In contrast, AD-MSCs were suitable for peri-transplantation immunosuppression. The proper combination of AD-MSCs, lymphodepletion and conventional immunosuppressants could consistently induce donor-specific tolerance to vascularized composite allotransplants (VCA). For each type of transplantation, optimizing the choice of therapeutics, timing, dose, and frequency of administration may be required. Future progress in the application of adipose-derived therapeutics to induce transplantation tolerance will be further benefited by continued research into their mechanisms of action and the development of standardized protocols for isolation methodologies, cell culture, and efficacy evaluation.

## 1 Introduction

Organ and tissue transplantation has emerged as a highly effective approach for patients with terminal illness or severe tissue defects; however, rejection caused by the immunological barrier between the donor and the recipient remains a significant problem. In most cases, this can be prevented or treated using combinations of immunosuppressants. However, long-term intake of these medications can potentially cause serious side effects such as organ failure due to drug toxicities and malignancies. A tremendous amount of research effort has focused on strategies to induce transplantation tolerance, which would enable transplant recipients to be free of the burden of immunosuppressants. To this end, therapy with cells exhibiting immunomodulatory properties, such as mesenchymal stromal cells (MSCs), has drawn considerable interest. Clinical studies have demonstrated the safety and feasibility of MSC therapy in transplantation (1). In kidney transplant patients, infused MSCs have allowed for a reduction in the dosage of the immunosuppressant tacrolimus (2, 3). In some living-donor kidney transplant patients, MSC therapy has been shown to facilitate the induction of transplantation tolerance (4, 5).

Although MSCs can be found in essentially all types of tissues, adipose tissue has been demonstrated to be a particularly good source of MSCs (6). The abundance of MSCs is approximately 500 times higher in adipose tissue than in bone marrow (BM) (7). Furthermore, adipose tissue is easily accessible due to its subcutaneous location and relatively rich volume, which are additional advantages of using it as a source of MSCs (8). In addition to adipose tissue-derived MSCs (AD-MSCs), stromal vascular fraction (SVF) and secretome have also been collected for use as therapeutics in various disease models, including transplantation. In this article, we present recent studies that have employed adipose-derived therapeutics in organ/tissue allotransplantation. The aim of this article is to stimulate interest in utilizing these therapeutic reagents in allotransplantation to promote allotransplant survival and explore their potential to achieve a consistent induction of tolerance.

## 2 Adipose tissue-derived therapeutics

**Figure 1:**
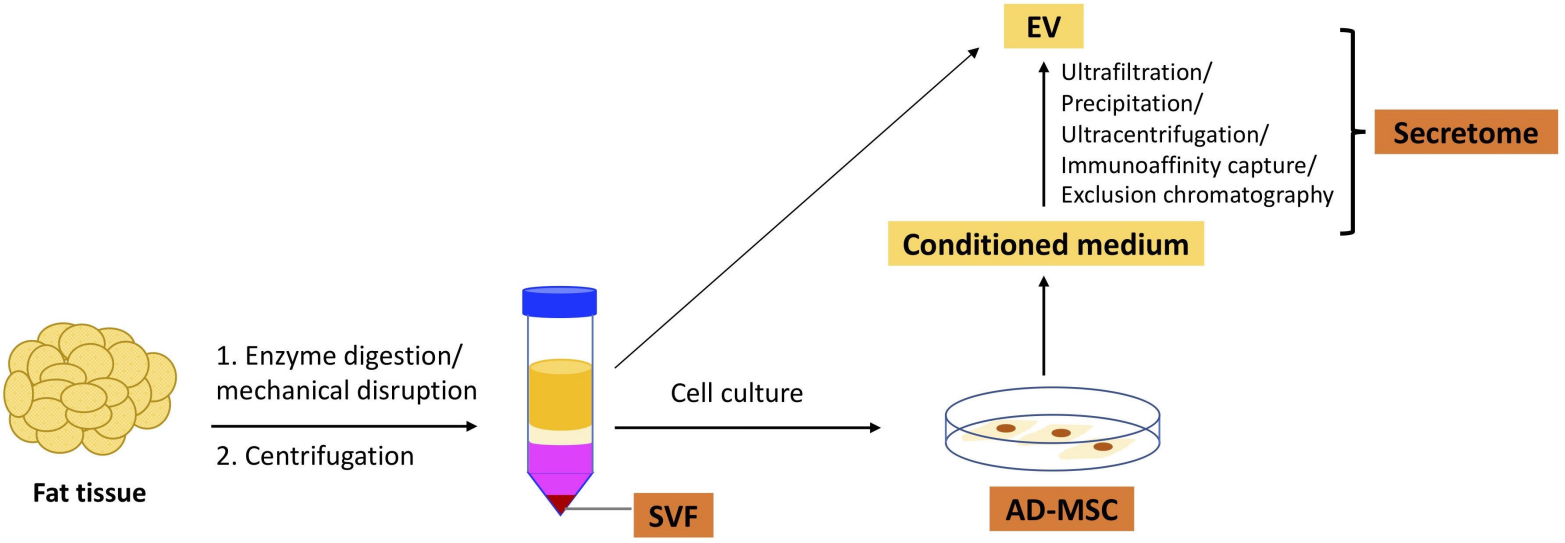
Isolation of adipose tissue-derived therapeutics from the fat tissue.

In this article, we will discuss SVF, AD-MSCs, and secretome. **Figure 1** illustrates the isolation of these three types of therapeutics from adipose tissue.

### 2.1 SVF

SVF is obtained by excluding mature adipocytes from adipose tissue through enzymatic digestion or mechanical disruption. It is characterized by multiple cell populations and generally includes 15–30% stromal cells (AD-MSCs), 10–20% endothelial cells (ECs) and endothelial progenitor cells (EPCs), 3–5% pericytes, and 25–45% hematopoietic cells (9, 10). Notably, SVF contains many regulatory-type leukocytes such as regulatory T cells (Tregs), regulatory B cells (Bregs), invariant natural killer (NK) T cells, and M2 macrophages (11–13). SVF has been shown to exhibit pro-angiogenic, anti-apoptotic, anti-inflammatory, and immunomodulatory capacities (14). Studies using a mouse hindlimb ischemia model have shown that SVF promotes wound healing (15) and vasculogenesis (16). SVF has also been demonstrated to ameliorate tubular injury in an acute kidney injury model by facilitating the proliferation of renal tubular epithelial cells (17). Andia et al. conducted a literature review and found that between 2010 and 2019, 73 clinical studies applied SVF in various contexts, such as arthritis, wound healing, erectile dysfunction, and cardiovascular and pulmonary diseases. The therapeutic efficacy of SVF has been demonstrated in many of these studies (18).

The therapeutic function of SVF can be derived from a specific cell population (such as AD-MSCs), or from crosstalk between SVF components. For example, reduced expression of costimulatory molecules and increased expression of transforming growth factor β (TGF-β) and interleukin-10 (IL-10) by M2 macrophages and AD-MSCs, respectively, enhanced the generation of Tregs, which then support the maintenance of M2 macrophages in SVF (19). The mutual paracrine interaction between AD-MSCs and EPCs, as well as the physical association between AD-MSCs and tubular structures formed by ECs, has been shown to contribute to the pro-angiogenic properties of SVF (20).

SVF has attracted considerable interest for clinical applications because it can be prepared at the point-of-care using commercially available devices which may be fully- or semi-automated, with established protocols (21, 22). SVF can be prepared within a few hours, making it a feasible option for emergencies (23). Other advantages include less manipulation and a lower risk of contamination (23). The therapeutic efficacy of SVF is greatly affected by the host condition due to the presence of multiple cell types and the lack of a culture or selection process between isolation and administration. For example, the reduction of vascular progenitor cells in adipose tissue with age leads to a decline in angiogenic potential and the capacity to establish a mature microcirculation of the isolated SVF (24, 25). Moreover, obesity results in increases in proinflammatory macrophages and CD8^+^ T cells, as well as a decrease in Tregs in adipose tissue, which subsequently affects SVF composition (26, 27). The preparation methods also influence the composition of SVF, with the percentage of AD-MSCs varying from 5% to 26% between mechanical and enzymatical preparation (28). The multiple-cell nature of SVF makes it potentially immunogenic, which is why autologous SVF has been preferred in most studies (29).

### 2.2 AD-MSCs

MSCs are heterogeneous progenitor cells with self-renewal and multilineage differentiation capacities (30). The criteria established by the International Society for Cell and Gene Therapy to define MSCs include plastic adherence; positive expression of CD73, CD90, and CD105; negative expression of human leukocyte antigen DR, hematopoietic and endothelial markers; and *in vitro* differentiation capacities into adipocyte, chondrocyte, and osteoblast lineages (31). By culturing SVF in an appropriate medium and onto culture dishes, AD-MSCs within the SVF can adhere, grow, and expand on the surface. Although AD-MSCs are less heterogeneous and better characterized than SVF, they require more time to culture and expand to reach substantial quantities. Cultured AD-MSCs are subject to risks of contamination, senescence (32, 33), and phenotypic changes, such as gradual disappearance of CD34 expression during *in vitro* culture (34, 35). The properties of AD-MSCs are also affected by cell culture parameters such as seeding density, growth media supplements such as fetal bovine serum, and environmental oxygen concentration (36). Therefore, the specific properties of MSCs can be enhanced by manipulating the culture conditions. For example, the addition of proinflammatory cytokines or reduction of environmental oxygen levels can enhance the immunomodulatory efficacy of AD-MSCs (37–39).

Numerous studies have demonstrated that AD-MSCs exhibit immunomodulatory functions. They are capable of suppressing the proliferation of effector T and B cells and impairing NK cell function and dendritic cell (DC) maturation, while enhancing the generation of Tregs, Bregs, and M2 macrophages (40–42). These actions are mainly mediated by soluble factors secreted by MSCs such as prostaglandin E2, TGF-β, IL-10, and indoleamine-2, 3-dioxygenase (IDO). Other secreted factors, including vascular endothelial growth factor (VEGF), hepatocyte growth factor (HGF), and angiopoietin-1, participate in angiogenesis and tissue repair. MSCs employ another mechanism to exert their function by transferring mitochondria to other cells. The transfer occurs through direct cell-cell contact, where healthy and functional mitochondria are transferred by MSCs to cells that need additional energy production. Do et al. reported that Tregs maintained robust FoxP3 expression and suppressive function even under proinflammatory conditions when they received mitochondria transferred from MSCs (43). Moreover, damaged cells can utilize the transferred mitochondria from MSCs to produce more energy and facilitate repair processes (44).

AD-MSCs are generally considered to have low immunogenicity due to the absence of costimulatory and major histocompatibility complex (MHC) class II molecules (45), and low level of MHC class I molecules (46). However, evidence has shown that MHC class I molecules play a role in evading NK cell-mediated cytotoxicity and thus the survival of MSCs in an allogeneic setting (47). Therefore, both autologous and allogeneic AD-MSCs can be used as therapeutic agents. These cells can be prepared in advance with careful characterization and preserved under appropriate conditions for later use. Furthermore, allogeneic AD-MSCs can be manufactured on a large scale, making them a readily available treatment option for various clinical conditions (40). To reduce donor-to-donor variations, pooled MSCs from several donors were used in a clinical trial for pediatric graft-versus-host disease, demonstrating superior therapeutic efficacy compared to MSCs from individual donors (48). Such practices may help to reduce the cost of MSC therapy and facilitate its clinical application.

### 2.3 Secretome

As mentioned previously, MSCs exert various functions through secreting paracrine factors, collectively known as the “secretome” (49). Apart from cultured MSCs, the secretome can also be collected from the adipose tissue, and its therapeutic efficacy has been demonstrated (50, 51). The secretome mainly consists of soluble proteins such as growth factors and cytokines, lipid, free nucleic acids, and extracellular vesicles (EV) (52). The multitude of biologically active factors and the potential to serve as “cell-free” therapeutics, which may offer a better safety profile and lower immunogenicity, have drawn significant research interest in the secretome, especially EVs (53).

EVs refer to particles naturally released from cells and contain lipid bilayers but lack functional nuclei (54). Soluble factors secreted by AD-MSCs can be encapsulated as cargo and transported by EVs, which release the cargo through endocytosis, membrane fusion, or specific molecular recognition when they reach target cells (55). The cargo content varies depending on the cell origin and culture conditions. For instance, Shin et al. performed a proteomic analysis and identified 265, 253, and over 400 proteins in EVs from AD-MSCs, BM-derived MSCs (BM-MSCs), and fetal (placenta and Wharton’s jelly) MSCs, respectively. Of these proteins, 181 were common among EVs of all origins (56). Systemic literature searches have characterized 591 proteins, 604 miRNAs, and 84 mRNA species from AD-MSC-derived EVs, indicating the richness of the biological information carried by EVs (57). Additionally, the content and function of EVs can be regulated by manipulating the culture conditions of AD-MSCs. Treatment with proinflammatory interferon gamma (IFN-γ) and tumor necrosis factor α (TNF-α) can alter the miRNA profiles in EVs, which can shift macrophages from M1 to M2 phenotype (39). Hypoxia-conditioned MSCs secrete EVs with potentiated immunosuppressive capabilities (58). EVs derived from human AD-MSCs have been shown to promote the proliferation and migration of cultured ECs, neovascularization, re-epithelialization, and wound closure when applied in a murine skin injury model (59). Additional *in vivo* studies have demonstrated that EVs are capable of enhancing nerve regeneration and offering liver and cardiac protection (60–62). Purification of EVs from physiological fluids or conditioned cell culture medium can be achieved using various methods such as ultracentrifugation, size-exclusion chromatography, ultrafiltration, immunoaffinity capture, and polymer precipitation (63). However, acquiring EVs with high yield and consistent biological activities remains challenging. New technologies, such as microfluidics, are being developed to overcome this impediment (64, 65).

The conditioned medium (CM) is another form of AD-MSC secretome that has been demonstrated to have therapeutic efficacy in many reports (49). For instance, CM of human AD-MSC has been shown to promote mouse liver regeneration (66) and facilitate wound healing in a murine skin injury model (67). To partially purify the CM, fractionation techniques such as ultrafiltration can be used to concentrate the bioactive factors (65, 66).

## 3 Roles of adipose tissue-derived therapeutics in allotransplantation

**Figure 2:**
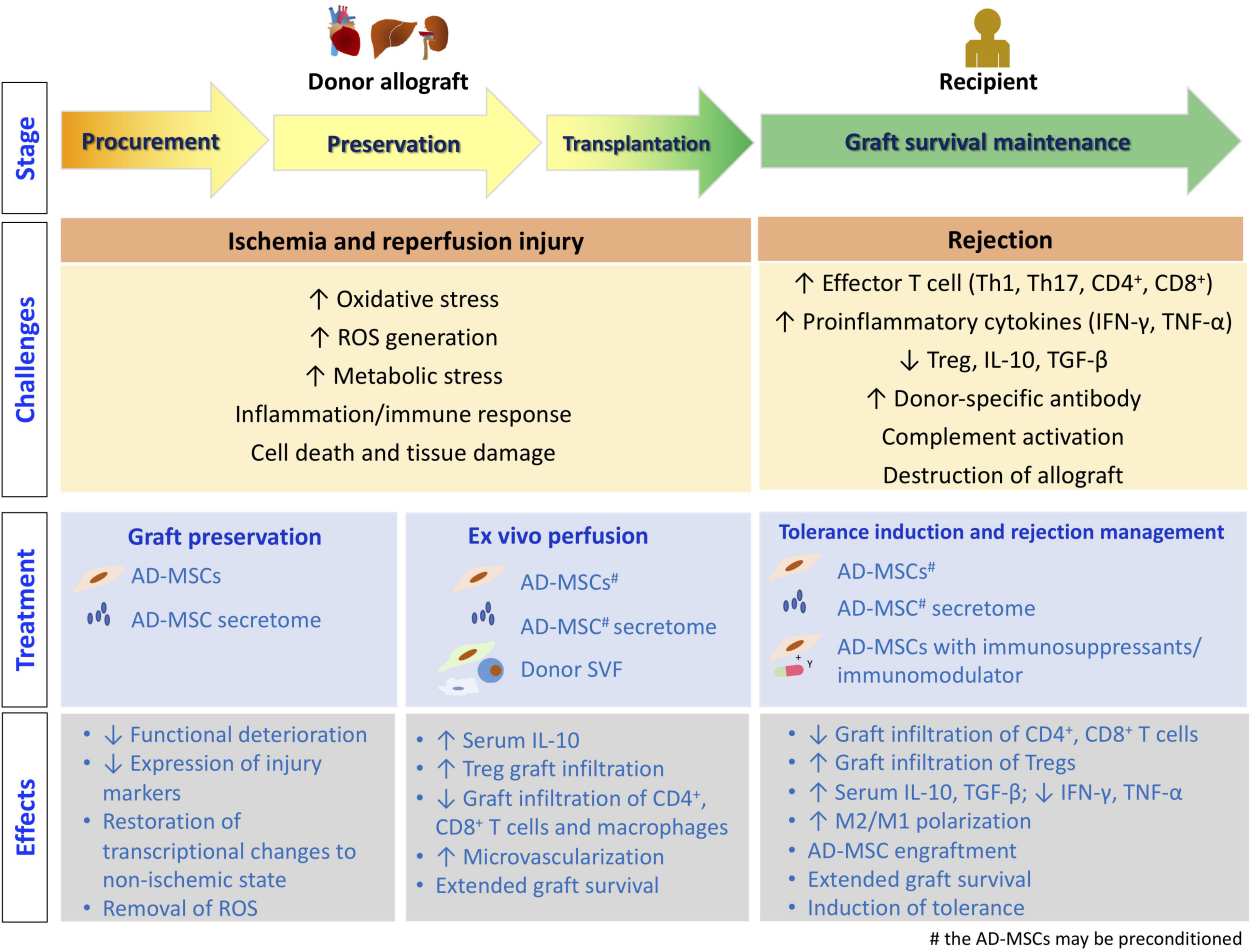
The stages of allotransplantation. The challenges encountered at each stage, along with the treatment strategies employing adipose tissue-derived therapeutics, are depicted. Additionally, the underlying molecular and cellular events are illustrated.

The process of organ/tissue allotransplantation can be divided into several stages, as illustrated in **Figure 2**. Allotransplants are inevitably subjected to ischemia–reperfusion injury (IRI) caused by hypoxemia and hypoperfusion during graft procurement, as well as oxidative stress and inflammatory events after revascularization in the recipients, before performing their physiological functions in the recipients after transplantation (68). After the transplantation surgery, managing the immune response induced by allogenicity between the donor and the recipient, such as treating rejection and inducting tolerance, is crucial for the success of allotransplantation. In this context, we discuss the current progress in adipose-derived therapeutics that can facilitate successful allotransplantation at different stages.

### 3.1 On the allotransplants

#### 3.1.1 Allotransplant preservation

IRI leads to the release of damage-associated molecular patterns, and facilitates leukocyte migration into the graft, complement activation, and allograft destruction. The intensity and duration of IRI are correlated with graft dysfunction and rejection after transplantation. Therefore, effective management of IRI may promote allograft survival and suppress rejection (69).

After procuring the allografts from the donor, they are perfused and stored in various preservation solutions to decrease their cellular metabolism and oxygen consumption (70). The preservation mode has evolved from a statically cold solution to normothermical machine perfusion (NMP), which allows better penetration of the solution, delivery of oxygen and nutrients, removal of toxic metabolites, and maintenance of normal cellular metabolism (71). However, tissue deterioration still occurs during organ preservation and transportation, which may prevent the procured grafts from being used. The tissue repair capacity of MSCs has been explored in this context. Kasahara et al. fractionated the AD-MSC CM and reported that addition of >50 kD or 10-30 kD fractions to the preservation solution of isolated islets during 24 hours of cold preservation can increase ATP production and restore the insulin-producing capabilities of the islets *in vitro* (72). When streptozotocin-induced diabetic mice were transplanted with islets that were previously preserved in the presence of these two AD-MSC secretome fractions, the recipients exhibited better glycemic control (73). Ellis et al. reported that functional deterioration of murine heart allografts was mitigated when AD-MSC secretome was added to the cold preservation solution (65). Wang et al. showed that miR-199-3p in the heart allograft was depleted during cold preservation, and the level can be recovered when the AD-MSC-derived EVs in the preservation solution transferred miR-199-3p to the graft (74). Ablation of superoxide dismutase 3 and/or catalase expression in AD-MSCs with siRNA attenuated the protective effects of AD-MSC secretome, suggesting that the removal of reactive oxygen species (ROS) generated during preservation is critical. Gene expression profiling studies showed that AD-MSC secretome partially restored the transcriptional changes induced by cold preservation, including mitochondrial dysfunction, apoptosis, and oxidative stress, to the non-ischemic (normal) state (75).

When kidney grafts underwent 2-3 hours of oxygenated hypotheric machine perfusion (HMP) followed by 7 hours of NMP, the addition of AD-MSCs during NMP suppressed the expression of injury markers such as neutrophil gelatinase-associated lipocalin and N-acetyl-β-d-glucosaminidase, while enhancing the expression of cytokines IL-8 and IL-6, which have proangiogenic and immunomodulatory effects (76). Subsequently, the authors tested another preservation protocol of 14 hours of oxygenated HMP followed by 4 hours of NMP for the kidney grafts and performed transplantation. Although approximately 5% of AD-MSCs were retained in the graft 2 weeks after transplantation, they had no effects on perfusion dynamics and did not improve graft function or survival (77). The authors suggested to optimize the dose and timing of AD-MSC administration, but the varied duration of HMP (2-3 hours versus 14 hours) and NMP (7 hours versus 4 hours) may have different effects on the graft and should be considered.

#### 3.1.2 Allotransplant pretreatment (ex vivo perfusion)

AD-MSCs and their secretome have been applied directly to allotransplants to mitigate injuries during procurement and storage before reperfusion is established in recipients. For example, when AD-MSCs were infused into the hindlimb allograft through the artery after procurement, allograft survival after transplantation was significantly prolonged from 15.5 days to 26 days (78). Hypoxia-preconditioned AD-MSCs had more pronounced effects and further extended graft survival to 32 days. Recipients of AD-MSC-infused allograft exhibited higher serum levels of IL-10 and increased infiltration of Tregs into the grafts than those without MSC infusion (78). Kato et al. found that kidney graft survival was significantly prolonged when AD-MSCs were administered intra-arterially to the kidneys before procurement. After transplantation, the AD-MSC-treated kidney grafts showed approximately a 50% reduction in CD4^+^ and CD8^+^ T cell infiltration, in addition to 3-fold induction of the anti-inflammatory factor TNF-stimulated gene 6. The infused AD-MSCs were identified in the graft glomeruli from postoperative day (POD) 1 to 3 and disappeared on POD 5 (79). However, the intra-arterial infusion of AD-MSCs into the kidney graft after warm ischemia and 16 hours of static cold storage did not mitigate the deterioration of the ischemia-induced glomerular infiltration rate or tissue fibrosis-related gene expression (80).

The mode of ex vivo infusion of AD-MSCs affects the outcome of allotransplantation. AD-MSC infusion into the lumen and subserosal of the small intestine allograft before reperfusion helped reduce the occurrence of acute rejection and significantly prolonged recipient survival, along with elevation of blood Tregs and TGF-β levels (81). However, intra-arterial infusion of AD-MSCs had no effect on the rejection of small intestine allografts (82). In a skin graft model, subcutaneous injection of AD-MSC-derived EVs into a graft that experienced 6 hours of ischemia before reperfusion reduced the inflammatory response and apoptosis. The area of blood perfusion and microvascular density measured 5 days after surgery were significantly improved with AD-MSC-EV administration. The beneficial effects on graft survival and neovascularization were significantly enhanced when the flap was treated with EVs derived from the H_2_O_2_-pretreated AD-MSCs (83).

Although ex vivo perfusion appears promising, caution should be exercised when administering high cell doses. Munk et al. reported that intra-arterial infusion of 10^7^ AD-MSCs was well tolerated during porcine renal transplantation, whereas the infusion of 10^8^ AD-MSCs caused serious adverse effects, including reduced renal perfusion and serious inflammatory reactions (84).

In addition to AD-MSCs, donor SVF was administered intra-arterially before reperfusion in a rat donation after circulatory death (DCD) kidney allotransplantation model. The recipients showed alleviated IRI and attenuated acute rejection. Enhanced microvascularization, elevated IDO expression and increased Treg infiltration were identified in the grafts. In contrast, reduced infiltration of CD8^+^ T cells and macrophages was observed (23, 85). Chen et al. compared the allografts infused with AD-MSCs and SVF prior to transplantation and reported that the SVF group had a significantly longer survival time than the AD-MSC group (35 versus 25.7 days), along with higher IL-10 and TGF-β levels and lower TNF-α levels in serum. Moreover, the SVF-infused graft had a higher density of CD31^+^ cells and vessels, suggesting that the pro-angiogenic effect of SVF is advantageous for graft survival (86).

In summary, current data have demonstrated that applying adipose-derived therapeutics to allografts can potentially promote graft function and survival after transplantation. Adding AD-MSC secretome to a cold preservation solution has been shown to improve graft function after transplantation (65, 72, 73). However, the benefits may be limited if the grafts undergo prolonged cold preservation before the application of AD-MSCs in NMP (77, 80). For ex vivo perfusion, SVF appears to be more effective than AD-MSCs, potentially due to its pro-angiogenic capacity (86). The choice of therapeutics, dose, timing, and application mode may need to be optimized based on the type of allotransplantation.

### 3.2 On the recipients

#### 3.2.1 Peri-transplantation immunosuppression/tolerance induction

The well-documented immunomodulatory capacities of MSCs have led to their use in many applications during the peri-transplantation period to mitigate the alloimmune responses elicited by the interaction between donor antigens and the recipient immune system after allotransplantation. Adipose-derived therapeutics have gradually gained recognition in this field. For example, among the preclinical studies summarized in **Table 1**, approximately one-third were published after 2020, including three with AD-MSC-derived secretome (91, 97, 115). As each study applied different variables, we group them into three subsections (3.2.1.1 to 3.2.1.3) for discussion.

**Table 1:** Preclinical studies with AD-MSCs and secretome for peri-transplantation immunosuppression.

Allograft type/ specifics	Species donor_recipient	AD-MSC or secretome^&^ origin_dose _ timing (route)	Immunosuppression	Allograft survival (MSC vs. control) (days)	*in vivo* molecular/cellular findings associated with AD-MSC/secretome administration^%^	Ref.
aorta	mice Balb/c_C57BL/6	syngenic_1x10^6 _ POD -1,1,7,14,21,28,35 (iv)	melatonin 200 mg/Kg POD -1 to 40	NR, endpoint at POD 40	- ↓ CAV - ↓ Th1, Th17, CD4^+^ Tmem, ↑ Treg, IL-10 - ↓ IFN-γ, TNF-α, IL-1β, IL-17, IL-6, MCP-1	(87)
heart	rat F344 to LEW	donor_1.2x10^6_ 3^rd^ hr after brain death (iv to donor); POD 1,3 (iv)	none	NR, endpoint at POD 5	- ↑ left ventricular ejection fraction (LVEF) - ↑ Tregs, IL-10, IL-34 - ↓ DNA damage, apoptosis, fibrosis - ↓ DC, T and macrophage infiltration, blood Ly6G^+^ cells, TNF-α, NF-κB, MMP-9, IL-6, MIP-1α	(88)
heart	mice Balb/c_C57BL/6	sFlg2 transfected recipient_1x10^6_ POD 1 (iv)	none	52 vs. 8.3	- ↓ TNF-α, NF-κB, MMP-9, IL-6, MIP-1α, - ↓ IFN-γ, TNF-α, IL-1α, IL-6, IL-12, ↑ IL-4, IL-10, TGF-β - ↑ M2/M1 macrophage polarization, Tregs	(89)
heart	mice Balb/c_C57BL/6	IL-35 transfected recipient_1x10^6_ POD 1 (iv)	none	17.5 vs. 6.2	- ↑ Treg - ↓ Th17, Th1/Th2 ratio,	(90)
heart	mice Balb/c_C57BL/6	IL-35 transfected recipient; or EV_ 10^6_POD 0-4 (iv)	none	24.3 (AD-MSC) vs. 21.8 (EV) vs. 6.7	- ↓ IL-17, ↑ Tregs and IL-10 - EV secretion blocker GW4869 partially blocked the effects of cells and EVs	(91)
heart	mice C57BL/6_Balb/c	donor_1x10^6_ POD -4 (iv)	MMF 160 mg/kg POD 0-7	33.9 vs. 16.8	- ↑ Th17 differentiation *via* PGE2-dependent induction of myeloid-derived immunosuppressive cells - MMF induced conversion of Th17 to Tregs	(92)
heart	mice C57BL/6_Balb/c	donor_5x10^6_ POD -4 (iv)	MMF 160 mg/kg POD 0-7	36 vs. 19	- the intact rather than heat-inactivated AD-MSCs prolonged graft survival when MMF is co-administered	(93)
heart	mice Balb/c_C57BL/6	poly(I:C) preconditioned recipient_5x10^5_ POD 1 (iv)	rapamycin 0.4mg/kg, POD 0,1,2,4,6,8,10,12,14,16	12.3 vs. 7	- ↑ Treg, Fgl2 expression and secretion - ↓ graft infiltration	(94)
islet	mice Balb/cA_C57BL/6J	recipient_2x10^5_ cotransplant w islet	none	13.6 vs. 1.2	- ↑ revascularization - ↓ non-fasting glucose level - ↓ CD4^+^, CD8^+^, CD68^+^ infiltration - ↓ islet mass required for reversal of diabetes	(95)
kidney/ orthotopic	rat BN_LEW	OX40L transfected recipient_2x10^6 _POD -4 (iv); 0 (intrarenal); 6 hr after surgery (iv)	none	14.2 vs. 6.2	- ↓ serum creatinine elevation and pathological changes (tubulitis, interstitial edema, parenchymal mononuclear cell infiltration) induced by transplantation - ↓ IFN-γ, ↑ TGF-β, IL-10, Foxp3 expression	(96)
kidney/ heterotopic	rat F344_LEW	recipient_1x10^6 cells or 1.4x10^9 EV_ POW 0,4,8 (iv); donor_1x10^6 cells or 1.4x10^9 EV_ POD 0 (iv)	none	- recipient: 77 (cell) vs. 57 (EV) vs. 58 - donor: 25 (cell) vs. 58 (EV) vs. 58	- EV did not promote graft survival - recipient AD-MSCs improved tubular damage, ↓ B, T, and NK cell infiltration - donor AD-MSCs accelerated end-stage kidney disease	(97)
liver	rat SD_Wistar	donor_2x10^6_ POD -7,3 (iv); in surgery(intraportal)	none	NR, endpoint at POD 7	- ↓ AST, ALT, TBIL, IL-2, ↑ IL-10 - alleviated graft rejection; ↓ apoptotic cells and infiltration	(98)
liver/ 50% reduced size	rat LEW_BN	recipient_2x10^6_after surgery (intraportal, iv)	none	24 vs. 13	- ↓ AST, ALT, TBIL and rejection index - ↓ IL-2, IL-17, higher IL-10, TGF-β and Tregs	(99)
liver/ from DCD donor	swine	allogeneic_1x10^7_2hr after reperfusion (intraportal)	none	5.4 vs. <1	- AD-MSC engraftment - ↓ apoptotic cells	(100)
lung	rat BN_LEW	autologous_1x10^6_after surgery (iv)	tacrolimus 0.5 mg/Kg POD 0-7	NR, endpoint at POD 7	- ↓ rejection score - ↑ HGH, and c-Met in DCs - AD-MSC engraftment	(101)
skin	mice CBA/J_C57BL/6	recipient or donor _5x10^5_POD 1 (ip)	none	17 (donor) vs. 12.5 (syngeneic) vs. 12	- ↑ degree of collagen orientation, neutrophil infiltration, ↑ VEGF and Foxp3 expression - ↑ Tregs in lymph nodes, IL-10, ↓ IL-17	(102)
skin	mice C57BL/6_Balb/c	xenogenic (human) 1x10^6, or 25x CM_POD 0 (iv)	none	23.9 (AD-MSC) vs. 19.6 (25x CM) vs. 9.3	- AD-MSC engraftment - hyporesponsiveness of recipient splenocytes to donor antigens - ↓ serum IL-6, graft TNF-α, IFN-γ, IL-10 - ↑ graft VEGF expression of the CM groups	(103)
skin	mice Balb/c_C57BL/6	recipient_1.5x10^5_POD 0 or 0,3,7 or 0,3,5,7,10 (ip)	none	>20 (all groups with cell infusion) vs. 12	- AD-MSC engraftment - ↓ TNF-α, IL-6, ↑ VEGF expression - ↓ lymphocyte infiltration	(104)
skin	mice Balb/c_C57BL/6	human_3x10^6 + donor BMC 5x10^5_POD 7 (iv)	α-CD4 and α-CD8 POD 0,2,5,7,14; busulfan 5mg/Kg POD 5	>200 vs. ~32.4^#^,	- sustained mixed chimerism at spleen and BM - clonal deletion at circulation and BM - ↑ donor-derived and total Tregs - tolerance induced (100% of AD-MSC group)	(105)
small bowel	rat BN_LEW	recipient_2x10^6_0 and 6h after surgery (iv)	none	18 vs. 11	- ↓ apoptotic cells in graft mucosae - ↑ IL-10, TGF-β, reduced IL-2 and IL-17 - ↑circulatory Tregs	(106)
VCA/ inferior epigastric flap	rat BN_LEW	human cells transfected CCR7_2x10^6_POD -1 (iv)	none	14.4 vs. 7.1	- ↓ IL-2, IL-6, IL-17, IFN-γ, ↑ IL-4, IL-10 - ↓ Th1/Th2, Th17/Treg in spleen, lymph node - changes of splenic protein profiles	(107)
VCA/ hindlimb	rat Wistar to SD	donor_1x10^5_ POD 0-3 (iv)	none	12 vs. 6.8	- ↓ mononuclear cell infiltration in graft - ↑ Foxp3^+^ cells in graft	(108)
VCA/ hindlimb	rat BN_LEW	donor_2x10^6_ POD 7,14,21 (iv)	ALS POD -4,1; CsA 10 mg/Kg POD 0-20	>150 vs. ~45^#^	- sustained peripheral chimerism - ↑ peripheral and graft Treg - ↑ blood IL-10, TGF-β1	(109)
VCA/ hindlimb	rat BN_LEW	donor_2x10^6_ POD 7,14,21 (iv)	ALS POD -4,1; CsA 10 mg/Kg POD 0-20	>150 vs. ~45^#^	- ↑ serum β2-glycoprotein, α1-macroglobulin, rat-albumin, and vitamin D-binding protein, and significantly lower haptoglobin by proteomic analysis	(110)
VCA/ hindlimb	rat BN_LEW	donor_1x10^6 or 5x10^6_POD 1 (iv)	ALS POD -4,1; FK506 0.5 mg/Kg POD 0-21	95 (1x10^6) vs. 58 (5x10^6) vs. 35	- transient blood chimerism on POD 28 - ↑ blood Tregs transiently - tolerance induced (47% of AD-MSC group)	(111)
VCA/ hindlimb	rat BN_LEW	donor_1x10^6_ POD 1, or 4, or 4,8,15 (iv)	ALS POD -4,1; FK506 0.5 mg/Kg POD 0-21	92 (POD 1) vs. 45 (POD 4) vs. 50 (POD 4, 8, 15) vs. 33	- ↑ Treg level by repetitive AD-MSCs - ↓ PBMC proliferation against donor antigens - ↓ intima/media ratio - tolerance induced (50% groups POD 1 and POD 4, 8, 15)	(112)
VCA/ hindlimb	rat BN_LEW	donor_1x10^6_ POD 2,4,7,14,28 (iv)	ALS POD 1,5 or cyclophosphamide POD 3; FK506 0.5 mg/Kg POD 0-14; CTLA4Ig POD 2,4,7	>120 (ALS) vs. 28 (cyclophosphamide) vs. 27.5	- tolerance induced (85% of the ALS group) - chimerism of B, monocytes and granulocytes appeared earlier than T cells - the tolerant graft skin had more Tregs - ↑ serum IL-1a, IL-2, TNF-α, IP-10, MCP-1, MCP-3 levels at rejecting recipients.	(113)
VCA/ hindlimb	rat BN_LEW	recipient_2x10^6_POD 7, 14,21 (iv)	ALS POD -4,1; FK506 0.5mg/Kg POD 0-20	NR	- ↑ CD45RA^+^FoxP3^+^ cells (Bregs); - ↓ C4d expression in graft	(114)
VCA/ hindlimb	rat BN_LEW	donor EV 200ug_POD 1 (iv)	ALS POD -4,1; FK506 0.5mg/Kg POD 0-20	58 vs. 32.5	- ↑ higher peripheral chimerism - ↓ peripheral CD4^+^ T and Th1 cells, ↑higher Treg and Tr1 cells	(115)
VCA/ HLOMC	rat BN_LEW	recipient_2x10^6_POD 1 (iv)	ALS POD -1,10; CsA 16 mg/Kg POD 0-10	>150 vs. 32	- ↑ circulatory Tregs - supplementing presurgical irradiation did not have additional benefits on graft survival - tolerance induced (66%)	(116)
VCA/ HLOMC	rat BN_LEW	recipient_2x10^6_POD 1,8,15 (iv)	ALS POD -1,10; CsA 16 mg/Kg POD 0-10	48 vs. 32	- ↑ peripheral Tregs and ↓ CD8^+^ in tolerant recipients - higher chimerism in CD161^+^ and CD4^+^ cells - no benefits with multiple AD-MSC doses - tolerance induced (40%)	(117)
VCA/ HLOMC	swine	autologous_ 1x10^6/Kg _ POW 0,1,2,3 (iv)	Irradiation (total body, intrathymus) POD -1; FK506 1 mg/Kg POD 0-14, 0.5 mg/kg POD 15-28	>28 weeks vs. 58.1	- ↓ rejection grade and lymphocyte infiltration - ↑ graft and circulatory Treg - ↑ serum TGF-β, ↓ TNF-α - AD-MSCs engraftment - 80% long-term survival	(118)

NR, not reported; CAV, chronic allograft vasculopathy; POD, postoperative day; POW, post operative week; AST, aspartate transaminase; ALT, alanine transaminase; TBIL, total bilirubin.

&, AD-MSCs were applied if not specified.

#, estimated median survival from the corresponding survival curve. %, ↑, increase in level; ↓, decrease in level.

##### 3.2.1.1 Single treatment of AD-MSC or secretome

AD-MSCs alone have been shown to slightly prolong the survival of transplanted kidney, skin, intestine, and hindlimb allografts (97, 100, 103, 104, 106, 108). The associated mechanisms included AD-MSC engraftment; increased circulatory levels of IL-10, TGF-β, and Tregs; suppressed T helper 1 (Th1) and T helper 17 (Th17) cells; decreased circulatory levels of TNF-α, IFN-γ, and IL-6; and reduced graft infiltration of lymphocytes. Some studies have reported benefits of improving graft function with AD-MSC administration (88, 98, 101). Most studies employed systemic administration through the intravenous or intraperitoneal route. However, direct infusion of AD-MSCs through the portal vein after transplantation of a liver graft procured from a DCD donor and in cold static storage for 4 hours rescued the liver from primary graft nonfunction and prolonged graft survival from less than 24 hours (no AD-MSC infusion) to more than 7 days (100). Intraportal administration of AD-MSCs also extended graft survival from 13 days to 26 days in a small-for-size liver transplantation model (99). Advantage of the proximity of AD-MSCs with the allograft was corroborated when AD-MSCs were transplanted together with the islet grafts and led to a significant extension of transplanted islet survival from 1.2 days to 13.6 days. However, no benefits were observed when the AD-MSCs were implanted on the contralateral side to the islet graft (95).

Lee et al. compared the effects of AD-MSCs with those of concentrated AD-MSC CM and found that concentrated CM was less effective than AD-MSCs in prolonging graft survival when administered after surgery but transiently induced VEGF expression, which implies a proangiogenic effect (103). Ramirez-Bajo et al. reported that AD-MSC-derived EVs did not provide benefits in prolonging graft survival in a heterotopic kidney transplantation model (97).

Notably, Yip et al. adopted a combinatorial approach in a heart transplantation model. They infused AD-MSCs into the donor 3 hours after induced brain death. Following transplantation, AD-MSCs were administered to the recipients on POD 1 and 3. The recipients showed fewer circulatory proinflammatory Ly6G^+^ cells and more Tregs. A lower degree of DNA damage and less infiltration of CD4^+^ T cells, DCs, and macrophages were observed in the heart allograft. The effects were less pronounced when cells were administered to either the donor or the recipient alone (88).

##### 3.2.1.2 Treatment with preconditioned AD-MSCs

The immunomodulatory effects of MSCs can be enhanced by preconditioning before their *in vivo* application (119, 120). Two approaches to preconditioning are typically used. First, MSCs can be cultured under modified conditions, such as exposure to reagents like IFN-γ and TNF-α, or hypoxic environments. Second, specific protein expression level can be adjusted by gene transfection of MSCs (37). Because Toll-like receptor 3 (TLR3) activation has been shown to enhance the anti-inflammatory capacity of MSCs, Bao et al. preincubated AD-MSCs with the TLR3 agonist poly(I:C) before infusion into heart transplant recipients. The poly(I:C)-pretreated AD-MSCs expressed and secreted higher levels of fibrinogen-like protein 2 (Fgl2), a Treg-secreted factor. Transplant survival was 10.2 and 12.3 days when the recipients were infused with AD-MSCs and poly(I:C)-pretreated AD-MSCs, respectively. The extended survival was associated with reduced lymphocyte infiltration into the graft and an increased percentage of splenic Tregs (94). Fgl2 has been shown to potentiate M2 macrophage polarization. AD-MSCs overexpressing soluble Fgl2 can migrate to the transplant site and induce a higher intragraft M2/M1 ratio, resulting in significantly prolonged graft survival (89). When overexpressing another Treg effector, IL-35, the administered AD-MSCs resulted in lower splenic Th17 levels and Th1/Th2 ratio, in addition to prolonging the graft survival from 6.2 days to 17.5 days, which was extended further to 24 days when the administration frequency was increased to 5 doses (90, 91). EVs from IL-35-transfected cells also prolonged the graft survival, which was only partially blocked by the IL-35 neutralizing antibody, suggesting the involvement of other factors in EVs. The capacity of AD-MSCs to suppress T cell proliferation was potentiated by overexpressing another costimulatory molecule, OX40Ig. When the OX40Ig-transfected AD-MSCs were administered to the recipients, graft survival was extended, along with reduced tissue damage in the transplanted kidney (96). AD-MSCs transfected with secondary lymphoid organ (SLO)-targeting CCR7 migrated to the SLO following *in vivo* administration to skin flap recipients. Transplant survival was prolonged in association with lower Th1/Th2 and Th17/Treg ratios within the spleen and lymph nodes (107).

##### 3.2.1.3 Effects of supplementary immunomodulators and immunosuppressants

The immunomodulatory effects of wild-type AD-MSCs can be potentiated by co-administration of immunomodulatory melatonin (87) or immunosuppressants (121). Obermajer et al. reported that AD-MSCs only prolonged graft survival when co-administered with the immunosuppressant mycophenolate mofetil (MMF) in a murine heart transplantation model. The authors found that the administered AD-MSCs induced Th17 generation, and the presence of MMF induced the conversion of Th17 to Tregs (92). These effects were abolished when AD-MSCs underwent heat inactivation, suggesting the requirement of intact cells (93). Interactions between MSCs and common immunosuppressants have been demonstrated in many studies. For example, cyclosporin A (CsA) has been shown to promote the immunomodulatory function and survival of MSCs (122). Conversely, MSCs potentiate the suppression of Th17 and Th1 by immunosuppressants (123). In a lung transplantation model, supplementary FK506 to AD-MSCs significantly ameliorated the severity of rejection and increased the production of hepatocyte growth factor (HGF) (101). Several studies have demonstrated the successful induction of donor-specific tolerance (111–113, 116, 117) or long-term graft survival (>120 days) (109, 110, 118) in vascularized composite allotransplantation (VCA) models, including transplantation of hindlimb and hindlimb osteomyocutaneous (HLOMC) grafts, with AD-MSC infusion supplemented with short-term (10–28 days) application of immunosuppressants and lymphodepletion reagents. The associated molecular/cellular changes included reduction in blood CD8^+^ cells and graft C4d deposits, and increased peripheral/spleen/graft Tregs (114). Serum levels of proinflammatory cytokines (TNF-α and IFN-γ) were decreased, whereas those of anti-inflammatory TGF-β and IL-10 were elevated in AD-MSC-infused VCA recipients (107, 109). Chen et al. reported that AD-MSC-derived exosomes extended hindlimb allograft survival in association with significantly higher levels of donor chimerism, Treg and type I regulatory T cells (115). Compared to the study by Plock et al., in which similar immunosuppression protocol was applied, the hindlimb allograft survival time was 92 and 58 days when the recipients were infused with intact AD-MSCs and AD-MSC-exosomes, respectively (112, 115). Although not a direct comparison, it appears that intact AD-MSCs perform better than AD-MSC-derived secretome in modulating the recipient immune response.

##### 3.2.1.4 Application of AD-MSCs for induction of clinical tolerance

In contrast to preclinical studies, the clinical application of AD-MSCs in organ/tissue allotransplantation is significantly lower, and available reports are from a single group. Vanikar et al. designed a protocol to infuse AD-MSCs, hematopoietic stem cells (HSC), peripheral blood stem cells (PBSCs), and donor-specific transfusion through portal circulation prior to living-donor kidney transplantation (LDRT) in recipients who were preconditioned with cyclophosphamide, total lymphoid irradiation, and rabbit anti-thymocyte globulin (rATG). They compared two groups of patients whose treatment differed only in the presence of AD-MSCs and showed that the group with AD-MSCs had better graft survival, lower serum creatine level, and fewer rejection episodes. In a group of 95 patients, 3 were weaned off immunosuppressants over the course of 7 years (4, 124). In a large-scale trial with 916 patients treated with the same protocol, pre-transplant infusion of stem cells resulted in a reduction of the triple-immunosuppressant regimen to two drugs in 71% (433/606) of patients or one drug in 3.9% (24/606) of patients (125). In a smaller group of patients who received AD-MSC/HSC/PBSC co-infusion 14 days prior to LDRT, followed by treatment with bortezomib, methylprednisolone, rATG, and rituximab between transplantation and POD 14, the authors reported that 5 of 10 patients did not require any conventional immunosuppressants and can be maintained with a low dose of prednisone (5mg/day) during the 6-year follow-up period (126). This protocol circumvented the use of conventional calcineurin inhibitors and promoted patient/graft survival by reducing the susceptibility to infection (126). A large-scale trial with different populations is necessary to further evaluate the efficacy of this protocol.

#### 3.2.2 Treatment of rejection

Clinically, rescuing the allotransplants from destruction when rejection occurs is a critical issue in transplantation. Although AD-MSCs have not been extensively explored for this purpose and no published reports are available, at least two clinical trials are currently underway to examine the efficacy of AD-MSCs in treating the rejection of lung and kidney transplants (NCT04714801 and NCT05456243, respectively). In contrast, completed clinical studies using BM-MSCs on chronic lung allograft rejection (127) or chronic antibody-mediated rejection of kidney transplants (128, 129) have demonstrated the safety, feasibility, and efficacy of MSC therapy for rejection. Additionally, umbilical cord MSCs have been shown to alleviate liver transplant damage during rejection (130). Given the promising results of BM-MSCs and umbilical cord MSCs for treating rejection, AD-MSCs have great potential to serve as an effective therapeutic option to rescue allotransplant rejection. However, additional investigation and validation are warranted.

## 4 Discussion

The adipose-derived therapeutics described in this article, namely SVFs, AD-MSCs, and secretome, have distinct advantages and disadvantages, as summarized in **Table 2**. Although SVFs are easy to isolate, their composition can vary depending on the host’s condition and the preparation methods. The efficacy of SVFs in allograft pretreatment has been demonstrated (85, 86). Secretome also performed well in allograft preservation and pretreatment (65, 72–75, 83). However, currently available data indicate that AD-MSCs are more effective than their secretome in managing the alloimmune response post-transplantation (97, 112, 115). Several studies have demonstrated the presence of AD-MSCs in the graft after systemic administration (100, 101, 103, 118), suggesting that the cellular responses elicited by AD-MSCs when encountering donor cells are beneficial for maintaining allograft survival in recipients. The timing and frequency of administration are also critical parameters. Plock et al. found that the recipient groups that were infused with AD-MSCs on POD 1 and 4 developed tolerance and rejected the graft by POD 60, respectively (112). As the recipients were treated with preconditioning reagents and immunosuppressants, the AD-MSCs may have encountered different states of the recipient immune system when they were infused at different timing. Furthermore, these reagents may influence MSC survival and competency. Notably, the group that received multiple cell administrations on POD 4, 8, and 15 developed transplantation tolerance, associated with a higher level of donor chimerism and sustained elevation of Tregs. Therefore, the interactions among the AD-MSCs, donor antigens, and recipient cells led to the cellular and molecular events that are crucial for determining the allograft outcome.

**Table 2:** Summary of adipose-derived therapeutics.

Type of therapeutics	Advantages	Disadvantages
SVF	• easy to isolate (point-of-care isolation) • no need of cell sorting and cell culture	• heterogenous cell composition • difficult to standardize and characterize • vary in composition and quantity depending on the host’s condition and isolation method
AD-MSC	• more defined characteristics • efficacy can be manipulated by preconditioning	• requirement of cell culture • potential phenotypic changes and senescence upon cell culture
secretome	• cell-free reagent • safe and low immunogenicity	• potential requirement of cell culture • isolation and characterization can be time-consuming

Based on the available literature, it is clear that administration of adipose-derived therapeutics alone is insufficient to induce transplantation tolerance and wean off immunosuppression usage for the recipients. However, transplantation tolerance has been induced in VCA models through the use of AD-MSCs along with preconditioning and short-term immunosuppression (131). A key characteristic of VCA is the inclusion of vascularized donor BM (VBM), which allows for the instant engraftment of donor bone marrow cells (BMCs) in a natural stromal-supporting microenvironment within recipients (132, 133). In allotransplantation, donor BMCs help establish transient or sustained chimerism, which may evoke central or peripheral clonal deletion and facilitate the induction of transplantation tolerance (134). Davis et al. successfully induced tolerance in a murine skin graft model by combining donor BMCs and AD-MSCs with CD4 and CD8 antibodies, as well as the antineoplastic reagent busulfan (105). The recipients showed sustained chimerism in the spleen and BM, and clonal deletion was evidient. Therefore, it is worth exploring a similar strategy that involves a combination of AD-MSCs, VBM/BMCs, lymphodepleting preconditioning, and short-term immunosuppression to induce tolerance in other types of allotransplants.

The effects of each therapeutic varied among studies. For example, donor-derived AD-MSCs performed worse than the recipient-derived cells in terms of kidney graft survival, but better in terms of skin graft survival (97, 102). Similarly, EVs were shown to prolong skin graft survival, but did not affect kidney graft survival (97, 103). Some of the observed variation may be due to inconsistent properties derived from variations in AD-MSC culture conditions or secretome collection. Furthermore, the efficacy of AD-MSCs and EVs may be improved by modifying the cell culture conditions. For instance, the preconditioning strategies mentioned earlier and three-dimensional (3D) spheroid cultures are worth further exploration. Previous studies reported that the 3D spheroids contain aggregates of cells and an intact extracellular matrix, closely simulating a natural environment compared to two-dimensional cultures. MSCs cultured in 3D have been shown with enhanced anti-inflammatory capacity (135, 136). EVs derived from 3D MSC cultures were also demonstrated to have better therapeutic efficacy than those derived from 2D cultures (137).

Overall, the use of SVFs, AD-MSCs, and EVs shows promise as an alternative to the lifelong intake of traditional immunosuppressive drugs. However, optimization of parameters such as administration timing, dose, and frequency is essential to maximize their benefits for each type of transplantation. Therefore, appropriate therapeutic agents should be selected for different purposes. For example, a combined strategy of applying SVF or secretome to pretreat allografts before transplantation to mitigate IRI, followed by utilizing the immunomodulatory function of AD-MSCs in the recipients, may be a feasible approach to improving transplantation survival. Future progress in the application of adipose-derived therapeutics in transplantation settings will be facilitated by continuing research into their mechanisms of action and the development of standardized protocols for isolation methodologies, AD-MSC culture, and efficacy evaluation.

## 5 Conclusion

Herein, we present recent studies that utilized the adipose-derived therapeutics including SVFs, AD-MSCs, and secretome to improve graft survival and induce transplantation tolerance in organ and tissue transplantation. These studies demonstrated their efficacy at different stages of allotransplantation. Although most of the evidence come from preclinical models, further standardization of techniques will help to allow a wider application of adipose-derived therapeutics for the induction of transplantation tolerance in clinical practice.

## Author contributions

H-YC conceived the study. H-YC and MRA wrote the manuscript. C-HL and F-CW reviewed and revised the manuscript. All authors contributed to the article and approved the submitted version.
